# Reinforced Universal Adhesive by Ribose Crosslinker: A Novel Strategy in Adhesive Dentistry

**DOI:** 10.3390/polym13050704

**Published:** 2021-02-26

**Authors:** Rim Bourgi, Umer Daood, Mohammed Nadeem Bijle, Amr Fawzy, Maroun Ghaleb, Louis Hardan

**Affiliations:** 1 Department of Restorative Dentistry, School of Dentistry, Saint-Joseph University, Beirut 1107 2180, Lebanon; rim.bourgi@net.usj.edu.lb (R.B.); maroun.ghaleb@net.usj.edu.lb (M.G.); louis.hardan@usj.edu.lb (L.H.); 2 Clinical Dentistry, Restorative Division, Faculty of Dentistry, International Medical University Kuala Lumpur, 126, Jalan Jalil Perkasa 19, Bukit Jalil, Wilayah Persekutuan, Kuala Lumpur 57000, Malaysia; 3 Paediatric Dentistry, Faculty of Dentistry, The University of Hong Kong, Hong Kong 999077, China; mnbijle@connect.hku.hk; 4 UWA Dental School, University of Western Australia, Nedlands, WA 6009, Australia; amr.fawzy@uwa.edu.au

**Keywords:** crosslinking, dentin, hybrid layer, ribose, universal adhesives

## Abstract

Enzymatic biodegradation of demineralized collagen fibrils could lead to the reduction of resin–dentin bond strength. Therefore, methods that provide protection to collagen fibrils appear to be a pragmatic solution to improve bond strength. Thus, the study’s aim was to investigate the effect of ribose (RB) on demineralized resin–dentin specimens in a modified universal adhesive. Dentin specimens were obtained, standardized and then bonded in vitro with a commercial multi-mode adhesive modified with 0, 0.5%, 1%, and 2% RB, restored with resin composite, and tested for micro-tensile bond strength (µTBS) after storage for 24 h in artificial saliva. Scanning electron microscopy (SEM) was performed to analyze resin–dentin interface. Contact angles were analyzed using a contact angle analyzer. Depth of penetration of adhesives and nanoleakage were assessed using micro-Raman spectroscopy and silver tracing. Molecular docking studies were carried out using Schrodinger small-molecule drug discovery suite 2019-4. Matrix metalloproteinases-2 (MMP-2) and cathepsin-K activities in RB-treated specimens were quantified using enzyme-linked immunosorbent assay (ELISA). The significance level was set at α = 0.05 for all statistical analyses. Incorporation of RB at 1% or 2% is of significant potential (*p* < 0.05) as it can be associated with improved wettability on dentin surfaces (0.5% had the lowest contact angle) as well as appreciable hybrid layer quality, and higher resin penetration. Improvement of the adhesive bond strength was shown when adding RB at 1% concentration to universal adhesive (*p* < 0.05). Modified adhesive increased the resistance of collagen degradation by inhibiting MMP-2 and cathepsin-K. A higher RB concentration was associated with improved results (*p* < 0.01). D-ribose showed favorable negative binding to collagen. In conclusion, universal adhesive using 1% or 2% RB helped in maintaining dentin collagen scaffold and proved to be successful in improving wettability, protease inhibition, and stability of demineralized dentin substrates. A more favorable substrate is created which, in turn, leads to a more stable dentin-adhesive bond. This could lead to more advantageous outcomes in a clinical scenario where a stable bond may result in longevity of the dental restoration.

## 1. Introduction

Significant contributions in the area of adhesive dentistry were instituted by the concept of the “etch-and-rinse” technique for bonding to dental substrates by Buonocore [1]. Since its development, different types of bonding agents and strategies have emerged which enhanced and facilitated dental bonding procedures [2]. Because enamel and dentin are different substrates, it is important to understand how they influence the adhesive performance [3]. Depending on the adhesive strategy used, the mineral component from dental substrates could be removed either partially (with acidic monomers in self-etch adhesives) or completely (by phosphoric acid in etch-and-rinse adhesives) [4]. Collagen matrix is then infiltrated with solvated monomers, thus creating the hybrid layer [5]. While bonding to enamel is a well-established technique [6], bonding to dentin, due to its structure, is considered challenging [7]. The durability of the resin–dentin bond relies on the quality of the hybrid layer [8]. However, its stability ultimately depends on each component’s resistance to degradation [9]. 

Recently, the introduction of “universal adhesives” to the market have urged clinicians to use them during clinical procedures due to their simplicity and versatility in the classical concept of dental bonding [10]. The concept provided more choices regarding the adhesion strategy; self-etch, total-etch or selective enamel-etch mode (total-etch on enamel and self-etch on dentin) [11,12]. However, a hurdle is presented when formulating universal adhesives, since water, which might be needed inside adhesive systems, may lead to the degradation of the chemical formulation of these systems, contributing to phase separation and reducing shelf life [13].

During bonding procedures, collagen fibers are exposed but might not be fully covered by the adhesive monomers since their penetration capacity is lower than the depth of demineralization causing adverse effects [7,8]. Demineralized collagen fibrils will be vulnerable to long-term hydrolytic or enzymatic degradation, leaving voids or demineralized nano-channels within the hybrid layer [14]. In order to enhance the quality of the resin–dentin interface, several studies focused to counteract enzymatic biodegradation by the use of matrix metalloproteinases (MMPs) inhibitors at the poorly resin-infiltrated hybrid layer [15,16,17,18,19,20,21] apart from other proposed clinically applicable strategies [22,23,24,25,26,27,28]. Since higher mechanical properties and lower biodegradation rates of collagen are desired, usage of collagen crosslinking agents in adhesive practices have gained interest [8,9,19,21]. They are successful in protecting collagen’s mechanical and chemical properties by enhancing intra- and inter-molecular crosslinks while providing stable collagen scaffolds [29,30,31,32,33]. 

Ribose (RB) is an organic compound classified as a monosaccharide (or simple sugar) that contains a carbonyl group and several hydroxyl groups (-OH) [34,35]. It is very active in non-enzymatic glycation and reacts rapidly with amino groups of proteins, to form a complex of products called the advanced glycation products (AGE) [36]. These AGEs are able to increase the matrix stiffness, decrease the solubility, and provide high enzymatic resistance to the crosslinked tissue [37,38]. Regarding the interaction between sugar and collagen, fewer studies in the field of dentistry are present; though an interaction between the amide group of collagens and the carbonyl groups of ribose exist, thus producing glycated derivatives [31,39]. The amino acid sequence of the bone matrix collagen is very rich in arginines and lysines, two key components for the AGE’s formation with sizeable space between each collagen fiber for the exposition of amino acids onto the surface of protein [40].

A previous study indicated that 1% ribose concentration resulted in long-term bond durability without disturbing the degree of polymerization [31]. However, further insights on the effect of ribose incorporated self-etch adhesive systems are yet to be explored relative to RB concentration’s dependent effect on bond strength, MMPs, and resin infiltrations. Therefore, the study’s objective was to assess the effect of adding different concentrations of ribose to a commercial multipurpose adhesive system in a self-etching mode on the dentinal bonding quality. The null hypotheses tested was that the modification of a universal adhesive with RB has no effect on the: (i) immediate bond strength, and (ii) inhibition of dentinal MMPs or cathepsin-K activities of the universal adhesive. In addition, the incorporation of ribose into the universal adhesive has no effect on the (iii) resin infiltration, or (iv) on dentin wettability properties of universal adhesives. 

## 2. Materials and Methods

Sound human molars free of fracture with no caries and resorptions (n = 140) were used after the approval of the Institutional Review Board of Saint-Joseph University (FMD-192; ref.#USJ-2019-154). Immediately after extraction, they were cleansed, then stored in 0.2% sodium azide solution at 4 °C for one month to inhibit microbial growth. The cross-linker used in the study was D-Ribose (C_5_H_10_O_5_, purity 99 wt %, Sigma-Aldrich, Ronkonkoma, NY, USA) and the universal adhesive system used was Prime&Bond active^TM^ (Dentsply DeTrey GmbH, Konstanz, Germany). The artificial saliva was prepared according to a method described in a previous study [41].

### 2.1. Preparation of RB-Modified Universal Adhesive 

Commercial universal adhesive (Prime&Bond active^TM^, Dentsply, 78467 Konstanz, Germany) was used and modified in three experimental groups. Unmodified adhesive was used as a control group. A total of 4 groups were obtained: Control (without modification), RB 0.5 (0.5% ribose + adhesive), RB 1 (1% ribose + adhesive), and RB 2 (2% ribose + adhesive). The ribose powder that was placed by a spatula was weighed using a precision analytic balance (Gold Bell Weigh System, Singapore, Singapore) and then placed into the adhesive bottle before being mixed. Thereafter, the mixture was vortexed on a continuous mode for 15 s. This step was done to yield a homogenous RB/solvent/monomer solution. To prevent spontaneous polymerization, the formulations were prepared in a dark room, under red light, at room temperature.

### 2.2. Specimen Preparation and Bonding Procedures

Twenty-eight teeth were prepared to evaluate the micro-tensile bond strength (n = 7). For each specimen, the occlusal superficial dentin surface was exposed using a slow-speed diamond saw (Isomet 1000; Buehler, Lake Bluff, IL, USA). The exposed dentin was then wet-grinded with 600 grit Silicon Carbide (SiC) polishing papers for 1 min, under a water spray, for smear layer standardization. Dentin specimens were randomly divided into four groups. Each specimen received one coat of universal adhesive applied in the self-etch mode, followed by a slight agitation for 20 s using micro brushes. Afterwards, preparation surfaces were gently air-dried using an air syringe for at least 5 s to evaporate excess solvents. Photo-activation was conducted at room temperature during 20 s by using a light curing unit equipped with a LED light (Dr’s Light Clever—Good Doctors, Seoul, South Korea) using an irradiance of 900 mW/cm^2^ (Table 1). 

Resin composite buildups were fabricated in two incremental layers of 2.0 mm each (A3 shade) using Filtek Z350 XT (3M ESPE, Minneapolis, MN, USA), and each layer was photopolymerized (Curing Light 2500, 3M ESPE, Minneapolis, MN, USA) for 20 s. After immersion in artificial saliva for 24 h at 37 °C, specimens were sectioned using a slow-speed diamond saw (Isomet; Buhler, Lake Bluff, IL, USA) to obtain resin–dentin beams (1.0 mm × 1.0 mm). The resin composite formed the upper half of the beam while the underlying dentin forming the lower half of the beam.

Four beams from the mid-coronal dentin were obtained from each tooth and kept moist until testing. 

### 2.3. Micro-Tensile Bond Strength

After storage in artificial saliva at 37 °C for 24 h, the beams were attached to a Geraldeli’s Jig device [42] using cyanoacrylate glue (Zapit; Dental Ventures of North America, Corona, CA, USA), and the µTBS tested in a universal testing machine (Model 4440, Instron, MA, New England, USA) at a crosshead speed of 1.0 mm/min with a 50 N load cell until failure. Thereafter, the cross-sectional area of the fractured specimens was measured using a Vernier caliper (CD-6BS; Mitutoyo, Tokyo, Japan). Bond strength (MPa) was obtained by dividing the maximal load [N] by the bonded surface area [mm^2^]. 

### 2.4. MMP-2 and Cathepsin-K Activity Determination 

Twenty-four teeth were used for this test. Dentin specimens were obtained by sectioning the teeth using a diamond disc perpendicularly along their long axis with a long-neck #4 spherical carbide burs (KG-Sorensen, Sao Paulo, Brazil) at a low speed, then stored in narrow mouth plastic bottles with screw caps (Z323012 Sigma, Detroit, MI, USA). Afterwards, specimens were frozen with liquid nitrogen, then pulverized into a fine powder using a steel mortar and pestle (Reimiller; Reggio Emilia, Turin, Italy). After that, dentin powder (1 g) was demineralized by applying 0.5 M EDTA (pH = 7.0), then followed by rinsing ten times with distilled water, drying, and randomly dividing them into four groups of 0.25 g each (n = 6). The dentin powder was then mixed with the different adhesive systems (Control, RB 0.5, RB 1, and RB 2) for 2 min. Thereafter, the dentin powder was resuspended in the extraction buffer (50 mM Tris-HCl, 0.2% Triton X-100, 5 mM CaCl_2_, and 100 mM NaCl) for 24 h in order to extract the proteases for quantification. Later, the vials were centrifuged at a temperature of 4 °C with a speed of 20,000 rpm for 30 min. The supernatants were then collected and dialyzed (30-kDa) overnight. Subsequently, they were lyophilized and frozen at −20 °C until analysis. ELISA (Human MMP-2 ELISA Kit, or Human CTSK/cathepsin-K ELISA Kit; Lifespan Biosciences, Seattle, WA, USA) was used to quantify the concentrations of MMP-2 and cathepsin-K in collected supernatants derived from the four groups at 7 and 14 days.

### 2.5. Morphology of Resin–Dentin Interface 

Specimens (n = 5/group) were restored using the same procedures used in the micro-tensile bond strength test and then sectioned perpendicular to the bonded surface to obtain resin–dentin slabs. Three slabs were obtained for each tooth. The slabs were polished with 600, 1200, 1500, and 2500-grit SiC papers (Carbimet; Buehler, Lake Bluff, IL, USA) and ultrasonically cleaned with distilled water for 10 min. The polished surfaces were dried by gentle blotting using fiber-free absorbent napkins (Kimwipes; Kimberly-Clark Professional, Roswell, GA, USA). After storing in artificial saliva at 37 °C for 24 h, the surfaces were etched with phosphoric acid gel for 15 s, rinsed with distilled water for 15 s, air-dried, and deproteinized by immersion in a 5.25% NaOCl solution for 20 min. Afterwards, they were washed with distilled water for 5 min. The specimens were then sequentially dried in ascending grades of ethanol (50%, 75%, 80%, 95%, and 100% ethanol) and sputter-coated with gold (Baltec SCD sputter, Scotia, NY, USA) for 120 s. The interfaces were examined by SEM (FEI XL30 FEG SEM; Philips, Tokyo, Japan) operated at an accelerating voltage of 10 kV at different magnifications. 

### 2.6. Micro-Raman Spectroscopy

Resin–dentin beams (n = 10) from each group were obtained and positioned in micro-Raman equipment (Senterra Raman microscope; Bruker Optics, Songdo, Yeonsu-Gu Incheon, South Korea). Spectra in the region of 3200 cm^−1^ to 400 cm^−1^ were obtained at the dentin–adhesive interface using a 500 μW single model laser with 785 nm wavelength. 

After zero calibration of the micro-Raman and by using the OPUS 6.5 spectral acquisition, the 785 nm single model laser focused at a power of <500 μW was used to collect spectra at a wave number region of 3200 cm^−1^ to 400 cm^−1^. The 100×/NA 0.9 objective lens used for the precision of the chemical data with a laser spot diameter of 1.0 μm was focused on the selected resin–dentin specimens placed over a glass slide and different locations were taken in 1 μm steps by using the computer-controlled x-y-z stage in order to induce the scattering effect of Raman.

Each scan was exposed for 60 s and each of the lines scanned across resin–dentin specimens were obtained from the central regions of the bonded specimen interface, starting in the dental composite region of the specimen (on the left), then across the resin–dentin adhesive interface, and ending onto the dentin side of the specimen (on the right). The measurements were done twice for each specimen in order to confirm the reproducibility of the technique. 

The intensity of peak 960 cm^−1^ (-PO_4_), 1667–1659 cm^−1^ (Amide I), and 1246–1243 cm^−1^ (Amide III) were taken as standards to analyze the changes in the components of dentin specimens. Peaks at 1640 cm^−1^ (C=C methacrylate groups) and at 1450 cm^−1^ (C-H alkyl group) were used for mapping the resin infiltration within the dentin.

### 2.7. Adhesive Contact Angle

Teeth were cut perpendicular to the long axial axis by means of a low-speed diamond saw (Accuton-50 machine; Struers, Copenhagen, Denmark) with water cooling to obtain dentin blocks with a 5.0 mm thickness (n = 4). The first cut was done on the occlusal third part of the crown in order to expose dentin and a second cut was achieved in the root 1 mm below the cemento-enamel junction. 

Each dentin block was further split into two semi-cylindrical halves. Commercial universal adhesive (Prime&Bond active^TM^; Dentsply, 78467 Konstanz, Germany) was used as the reference material for contact angle measurements. A drop (2 μL) of each of the different groups was applied into the dentin and the contact angle analyzer (Dental Simulation Lab; IMU Laboratory; Chuo-ku, Tokyo, Japan) was then used to record the profile of the droplet. After processing by a free software ImageJ using the tools contact angle plugin, the measurement was obtained. Accordingly, the program can fit the profile of the drop, placed on the dentin surface, and calculated the contact angle by using ellipse/sphere approximation. 

### 2.8. Molecular Docking Simulations 

Molecular docking reports were performed by using Schrödinger small-molecule drug discovery suite 2019-4 [43]. Crystal structures of collagen (PDB ID: 6A0C) were downloaded from the Research Collaboratory for Structural Bioinformatics Protein Data Bank (http://www.pdb.org, accessed on 30 December 2020) [44]. Throughout the protein preparation process, water molecules with less than three hydrogen bonds were removed. Hydrogen bonds (equivalent to pH 7.0) and missing side chain atoms and loops in the protein structure were added, followed by energy minimization by means of OPLS 2005 force field [44,45]. The binding site of collagen was detected using “binding site detection” function in Schrodinger 2019-4 suite. The 3D chemical structure of D-ribose was drawn using a software called Maestro 11.8; and 3D structures were prepared using Ligprep module; OPLS 2005 force field was employed to create the low-energy conformers. Low energy conformations of the compounds were docked into the binding site by using an extra precision (XP) mode [45].

### 2.9. Nanoleakage Analysis

Twelve teeth (n = 3) were used for evaluating nanoleakage amongst the resin–dentin interface specimens of different groups. After performing the bonding procedure as mentioned earlier, the teeth were kept in artificial saliva for 24 h for immediate evaluation. Nail varnish was applied 1.0 mm away from the bonded interface covering the entire surface except the resin–dentin bonded area. The specimens were then immersed in 50 wt % of ammonical silver nitrate (pH = 9.5) solution for 24 h. After that, the specimens were rinsed with deionized water and placed in a photo developing solution (TMAX Liquid Film Developer; Kodak, Warwick, RI 02887, England) for 8 h under fluorescent light. Once the specimens were removed from the photo developing solution, the specimens were again washed using deionized water, and wet polished with diamond pastes (3 µm; Buehler Ltd, Lake Bluff, IL, USA) using a polishing cloth. The specimens were then ultrasonically cleaned for 15 min, dried for 24 h, mounted on stubs, and coated with carbon. Resin–dentin interface was evaluated using SEM at 15 kV operated in a back-scattered mode. Around twenty images were taken from each group at 500× magnification, and silver deposition was assessed by two observers. Nanoleakage silver uptake was evaluated using the score devised by Saboia et al.: 0: no nanoleakage; 1: <25% nanoleakage; 2: 25 ≤ 50% nanoleakage; 3: 50 ≤ 75% nanoleakage; and 4: >75% nanoleakage [46].

### 2.10. Statistical Analysis

Analysis was performed using a statistical software program (SPSS v. 25.0; IBM Statistics, Chicago, IL, USA). The normality distribution of the contact angle and μTBS were assessed using Shapiro–Wilk tests. Levene test was used to analyze homogeneity of the variance. One-way analysis of variance (ANOVA) followed by Tukey’s honestly significant difference (HSD)post -hoc comparison tests were performed to compare the contact angle and the μTBS between groups. Dentinal MMP-2 and cathepsin-K concentrations were distinctly analyzed using two-way ANOVA. Concerning nanoleakage evaluation, the interobserver and the intraobserver agreement were evaluated through the weighted Kappa (kw) statistics. The levels of significance for all tests were set at α = 0.05. The sample size for analysis of specimens are derived (α = 0.05), keeping the power of study equal to 90% and level of significance equal to 5%.

## 3. Results

### 3.1. Micro-Tensile Bond Strength

The results of micro-tensile bond strength for every single group at 24 h are presented in Table 2. One-way ANOVA conveyed that factors “various concentrations (F = 47.4, *p* < 0.05)” of ribose significantly affected bond strength analysis. Bond strength value decreased significantly (*p* < 0.05) at baseline as the concentration of ribose rose to 2% (31.31 ± 6.7). All bond strength groups exhibited significant variations when compared to the control group (36.21 ± 13.8) with a significant increase in 1% ribose groups (39.44 ± 7.7). The bond strength showed a drop in 0.5% ribose groups but had no negative effect on the immediate bond strength of the groups. 

### 3.2. MMP-2 and Cathepsin-K Activity Determination 

Quantities of MMP-2 and C-terminal peptide (CTX) released after incubation of specimens for 7/14 days correspondingly are summarized in Table 2. Both factors, modification concentration (RB) and time, had significantly affected CTX release. The interaction of these two factors was similarly significant (*p* < 0.05). The results confirm that the 2% RB-treated specimens showed, in demineralized dentin, a significant decrease in cathepsin-K and MMP-2 activities in comparison to control, 0.5% and 1% RB specimens correspondingly over a period of 7 and 14 days. The significant differences were found in all specimens treated with RB as the cathepsin-K and MMP-2 decreased with a rise in ribose concentration (*p* < 0.05). There was a significant increase of proteases seen in control specimens between 7 to 14 days. 

### 3.3. Morphology of Resin–Dentin Interface 

**Figure 1:**
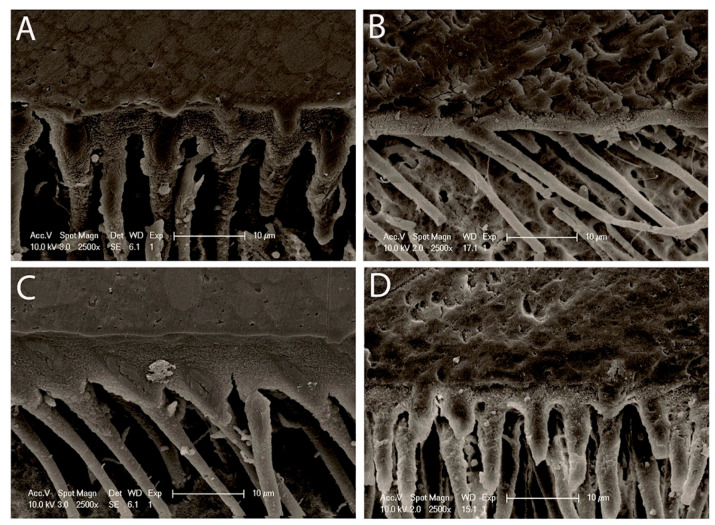
Representative SEM micrographs of the resin–dentin interface is shown from different bio-modification procedures. (**A**) Well-formed hybrid layer within the resin–dentin interface produced by Prime&Bond active^TM^ applied on self-etch mode and a regular cylindrical shape exhibiting a rough texture on top of the resin tags; (**B**) The 0.5% RB-modified adhesive specimens showed a thinner hybrid layer; (**C**) Relatively thicker and textured hybrid layer with long well-formed resin tags could be seen with the 1% RB-modified adhesive specimens; (**D**) Well-defined uniform hybrid layer and with well-formed branched resin tags could be observed with 2% RB-modified adhesive. HL: hybrid layer, RT: resin tags.

Figure 1 shows a view of resin–dentin interface of all the specimens, whether treated or untreated with RB, 24 h after immersion in artificial saliva. The hybrid layer or inter-diffusion layer, along with resin tags, is noticed in every bonded specimen. The thickness of the inter-diffusion layer and the length of the resin tags displayed a perceptible variation with respect to the % of RB added to the adhesive. A well-formed hybrid layer with some gaps within the resin–dentin interface produced by Prime&Bond active^TM^ (Dentsply DeTrey GmbH, Konstanz, Germany) applied on self-etch mode and a regular cylindrical shape demonstrating a rough surface over the resin tags (Figure 1A). The 0.5% RB-modified adhesive specimens presented a very thin hybrid layer with an absence of regular resin tags (Figure 1B). Additionally, the considerable size of the resin tags with several lateral branches and a clear, intact, and adequate hybrid layer are emphasized with 1% RB-modified adhesive specimens (Figure 1C). In contrast, intact hybrid layer for the 2% RB-modified adhesive and shapely resin tags can be witnessed (Figure 1D). Furthermore, the interface of the adhesive with the superimposing resin composite was undamaged and lacked any holes for the 0.5% and 2% RB-modified adhesive.

### 3.4. Micro-Raman Spectroscopy

**Figure 2:**
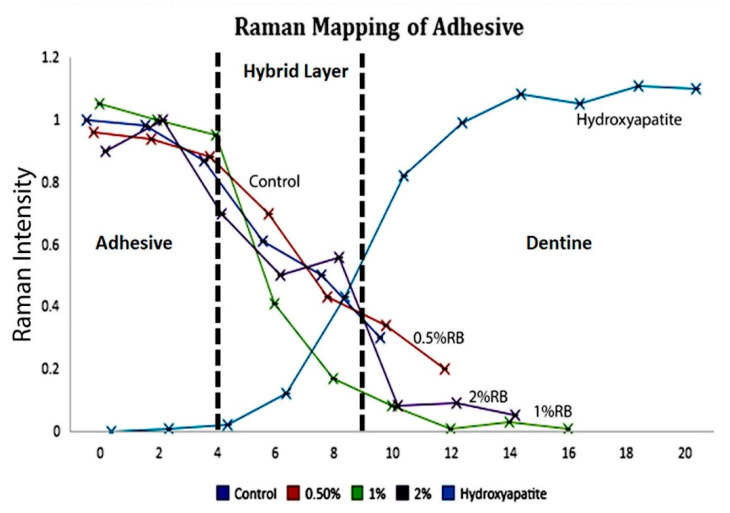
Representative line map (scans) across resin–dentin interface of different control and RB-modified adhesive specimens are shown. The spectral contribution is recorded at 960 cm^−1^ (hydroxyapatite) and 1450 cm^−1^ (C-H alkyl) intensities representing the penetration of different adhesives.

At 1 μm across the interface between resin and dentin, the micro-Raman spectroscopy in all four groups is presented in Figure 2. The Raman bands examined at 960 cm^−1^ (P–O peak) are linked to phosphate vibrations of hydroxyapatite, while the organic constituents inside the inter-diffusion layer and the adhesive resin were measured using 1450 cm^−1^ C-H alkyl group. Because of the uneven surface, variations were perceived through the interface in the hydroxyapatite dispersal. From the line maps, an ongoing decline in C-H band was seen in the area of 10 μm for the control group, while the depths obtained for the specimens treated with diverse concentrations of ribose were as follows: 0.5% RB-modified adhesive specimens, 12 μm; 1% RB-modified adhesive specimens, 16 μm and 2% RB-modified adhesive specimens, 14.2 μm. These outcomes implied an ample penetration of 1% RB-modified adhesive specimens across the hybrid layer and the least penetration seen with a full disassociation of Raman spectrum at the regions of the C-H band for the control group. The data obtained from micro-Raman implied the existence of a complex interaction between the resin and the resin–dentin specimens. The level of adhesive penetration was superior in the initial micrometers inside the interfacial region.

### 3.5. Adhesive Contact Angle

**Figure 3:**
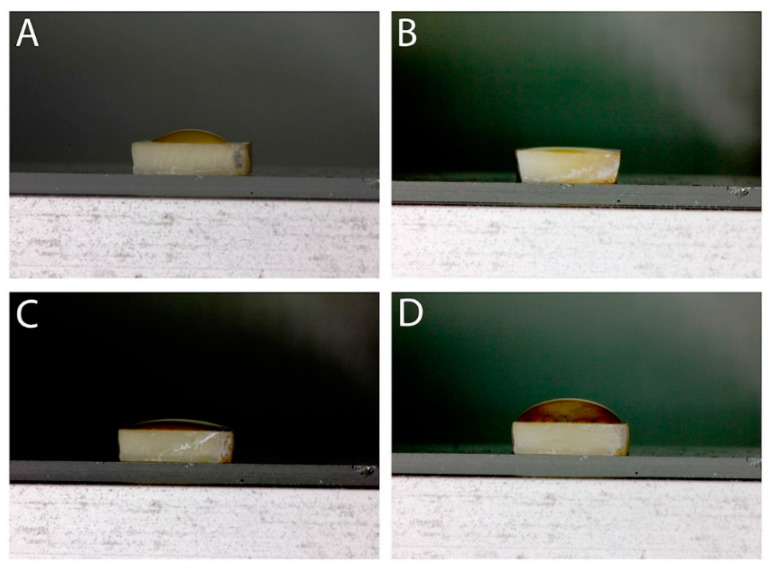
Representative images of contact angle of different adhesives; The adhesive drop is shown in (**A**) control; (**B**) 0.5% RB; (**C**) 1% RB and (**D**) 2% RB. All images captured were taken 5 min after their placement on the dentin surface. The placement of the adhesive quantifies the intrinsic aptitude of the adhesive liquid to spread on a flat, undeformable, and homogeneous solid dentin surface.

The mean contact angle was significantly different between the four groups. According to Tukey post hoc tests, it was significantly lower in specimens bonded with 0.5% RB-modified universal adhesive, followed by specimens bonded with 1% RB and 2% RB (*p* < 0.05), correspondingly. The mean contact angle was significantly higher in the treated control group. Results of the mean contact angle are shown in Table 2 and Figure 3.

### 3.6. Molecular Docking Simulations 

**Figure 4:**
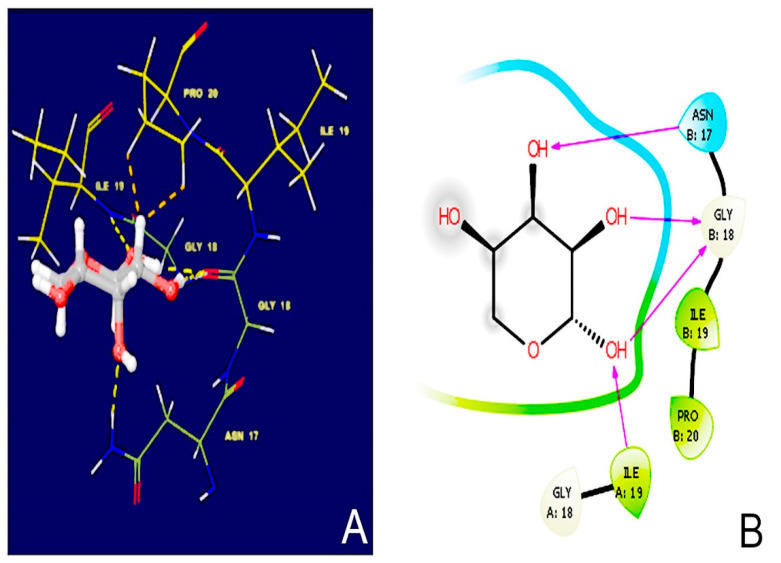
Results of molecular docking simulation of RB on crystal structure of collagen indicating a complex representing a predicted interaction mode of crosslinking. The structure was generated from molecular coordinates from the Protein Data Bank, PDB ID. Subset proposed chemical formula with molecular docking in (**A**) 3D and (**B**) 2D mode. The docking shown in this figure is typically performed based on the known compounds. The polar capabilities of ribose have enabled it to form charge–charge interactions that can insert with the binding pocket of collagen. Interactions that occur more than 5.0% of the simulation time in the selected trajectory (0.00 through 100.00 ns) are shown.

D-ribose binds to collagen (PDB ID: 6A0C) through: Hydrogen bonding interactions with asparagine (B17), glycine (B18) and isoleucine (B19); and Hydrophobic interactions with isoleucine (A19) and proline (B20). The docking score is accurately calculated as −4.384 Kcal/mole. The binding pose (3D and 2D), along with the key amino acid that participated in the binding process of D-ribose, are shown in Figure 4.

### 3.7. Nanoleakage Analysis

**Figure 5:**
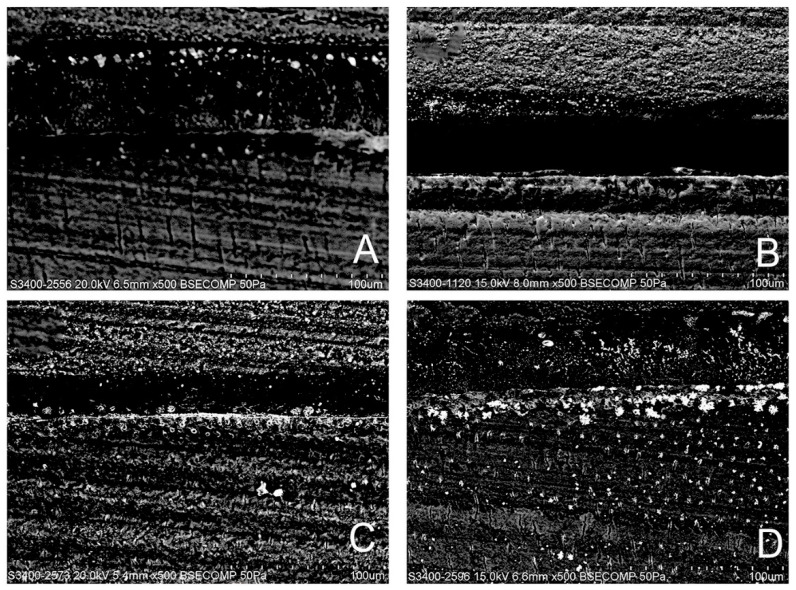
The distribution of nanoleakage scores of different groups is depicted in SEM images. (**A**) Control; (**B**) 1% RB; (**C**) 0.5% RB, and (**D**) 2% RB. The (**A**) control specimens showed thick adhesive layer with minimal silver deposition and no reticular silver depositions in (**B**) 1%RB specimens. Some nanoleakage expression was observed in 0.5% RB adhesive specimens with substantial silver deposition along dentin–resin interface and extended into the hybrid layer perceived in (**D**) 2%RB specimens.

Some gaps could be observed for the control and 1% RB groups (data not shown). The weighted Kappa for intraobserver and interobserver reproducibility surpassed the 0.70 cutoff, with a mean value of 0.86, signifying nearly complete reproducibility. The distribution of nanoleakage scores of diverse groups is depicted in Table 3 and Figure 5. The Cochran–Mantel–Haenszel technique was utilized in order to test for significant change among every pair of modified groups at baseline. For control and 2% RB groups, there was a lack of a significant variation in nanoleakage score at baseline (*p* > 0.05); whereas a significant variation in nanoleakage score was detected in 0.5% and 1% RB groups (*p* < 0.05).

## 4. Discussion

Numerous in vitro findings support the hypothesis that RB glycation has a crucial role in altering the mechanical characteristics of the bone [47,48]. A slight concentration of RB (30 mM) has a noticeable impact on the intermolecular crosslinking of collagen. Likewise, glucose-modified gelatin/collagen matrix proved to be a harmless and efficient biomaterial with exceptional biocompatibility [37]. By adopting the glycation method, this procedure has been suggested in order to enhance collagen crosslinking in dentin [31]. Having said that, the stability of the collagen matrix through pentosidine forming crosslinks between lysine and arginine residues in collagen might be affected by using ribose crosslinker [35,46]. This was anticipated to decelerate the pathological dentin caries progression via enhancement of the enzymatic degradation resistance [31]. 

A study done by Daood et al. in 2018 displayed, in vitro, a higher bond strength value when using an experimental ethanol-based two-step-etch-and-rinse adhesive modified by ribose at 1%. The results suggested that ribose can reinforce collagen and could open a new avenue in the era of collagen crosslinking [31]. In the current study, we have modified a universal adhesive Prime&Bond active^TM^ (Dentsply, 78467 Konstanz, Germany) with ribose at 0%, 0.5%, 1%, and 2%, correspondingly. Our study results showed that all bond strength groups presented significant deviations when placed in comparison with the control group (36.21 ± 13.8) with significant increase in 1% ribose groups (39.44 ± 7.7). This finding conforms with the previous study mentioned above, which used a different adhesion strategy. Accordingly, the primary null hypothesis stating that RB-modified specimens do not affect the micro-tensile bond strength after 24 h can be overruled. A noteworthy discovery deduced from this analysis is that the bond strength has decreased due to an increased concentration of ribose from 1 to 2%. This could be explained due to AGE formation and its effects were witnessed in a fashion reliant on dosage [49]. Moreover, the improvement could be linked to the presence of an increased number of intermolecular and/or intramolecular bonds within type I collagen at 1% ribose groups [31,50]. Suitably, a deleterious impact on the biochemical and biomechanical characteristics of the collagen network could be detected [31]. As stated before, the accumulation of AGE in the matrix of the bone is believed to hinder collagen fibril deformation (a brittle effect), and this energy dissipation loss leads to the reduction of fracture resistance of the bone [51]. Collagen becomes less soluble and their fibrillogenesis time increases [49].

Our study results showed that 1% RB adhesive specimens might be perceived as a minimum threshold level which significantly (*p* < 0.001) minimized the activities of both enzymes tested, thus rejecting the second null hypothesis. It was found that 2% RB-modified universal adhesive presents the least activity of MMP-2 and cathepsin-K. Our experiments clearly indicate that ribose glycation inhibits MMP-2 and cathepsin-K. A possible mechanism of this inhibition could be the potential formation of AGEs on lysine and/or arginine residues within a single collagen molecule as well as between two adjacent molecules [52]. In the case of collagen, the formation of cross-links causes bridges that stiffen the collagen molecules and increases their resistance to proteolytic enzymes, thus affecting matrix remodeling [53]. This results in increased tissue stiffness [53,54]. These experiments evidently denote that the AGEs possess the potential to mask the collagenase recognition sites [54] and could be considered to have an inhibitory effect on the degradation phenomena. In this study, resin–dentin interface of the specimens was analyzed for morphological stability. Prime&Bond active^TM^ with pH-2.5 [55] was used in this study in a self-etch strategy. The hybrid layer is thin (0.5–1.5 μm for mild or moderate self-etch adhesives) compared to that which can be formed after phosphoric acid etching, which is more acidic (5 μm for etch-and-rinse adhesives) [56]. The 0.5% RB-modified universal adhesive specimens exhibited a hybrid layer which was very thin, similar to the non-modified group, but lacked well-formed resin tags. Contrary to the previously mentioned groups, 1% and 2% showed a relatively adequate and highly textured hybrid layer with shapely and well-formed resin tags, indicating good resin infiltration, and numerous small lateral branches indicating a possible supplementary retention. Furthermore, the adhesive surface with the superimposing resin composite was shown to be whole and lacking any voids for the 0.5% and 2% RB-modified adhesive, while some gaps could be observed for the control and 1% RB groups. One should bear in mind that ribose enhances the nucleation kinetics within collagen favoring adhesion to protein [57], along with protein hydrophobicity [58]. However, ribose presents a level of saturation, as observed in our study; for control and 2% RB groups, a lack of significant variation in nanoleakage score was observed at baseline (*p* > 0.05); contrary to the 0.5% and 1% RB groups where significant variations in nanoleakage score were found (*p* < 0.05). Our experiments were conducted to determine the optimum concentration of ribose and the crosslinking reaction with respect to time for the physical and structural stability to lack any unfavorable response. Ribose with viable biocompatibility can be employed in clinical trials to improve the strength of dentinal collagen, as glycated samples preserve the capacity of material to store energy [59].

Based on the findings of this experiment, 1% RB adhesive specimens displayed higher amounts of resin penetration in comparison to the control, 0.5% and 2% modified adhesive specimens, which subsequently indicated a higher polymerization in decalcified dentin. Consequently, these results might suggest that higher penetration values (1%) activate RB and the photo-initiator simultaneously inside the adhesive, justifying and explaining the adequate depth of cure. Accordingly, the third null hypothesis can be rejected since the incorporation of ribose affected the penetration of resin inside the modified adhesive. A specific Raman peak at 1450 cm^−1^ is linked to the C-H band presented in most monomers and is representative for the adhesive penetration inside dentin specimens [60].

For optimum adhesion, an appropriate adhesive system with a good spreading capacity and a lower contact angle is necessary [61]. There was a statistical difference (*p* < 0.05) in the contact angle analysis between the 0.5% RB-modified specimens and all the other experimental groups. Thus, the fourth null hypothesis that ribose treatment modification exhibits no effect on the dentin wettability is rejected. In general, dissolving sugar in water yields a higher viscosity which, in turn, means that the spreading capacity of the adhesive is expected to decrease when modified with higher ribose concentration [62]. Therefore, the spreading capacity of adhesive increased with decreased concentration of ribose (1%). This can be interpreted by the interaction of ribose with the adhesive and, as a result, altering its composition and reducing its viscosity. Ribose dissolves easily in water, as it contains many polar hydroxyl groups (OH), which bond hydrogen with water molecules [34,37]. Presumably, ribose with smaller concentration may increase wettability, hence, facilitating better adhesive distribution on dentin surface.

Knowing that stability of collagen has always been a challenge for adhesion, researchers have franticly focused to improve the mechanical stability of this scaffold by protecting them using different crosslinking agents [21,29,30,31,32,33,37]. However, controlling pre-treatment procedures is a bit difficult and this makes it harder to determine the crosslinking effect [63]; on the other hand, rinsing of etching agent might have an unfavorable effect on dentinal crosslinkers. Consequently, incorporating RB inside an adhesive system provides a solution for collagen crosslinking within the organic matrix of dentin, thus abstaining from any pre-treatment techniques. Hence, this new universal adhesive formulation might possess significant potential in facilitating resin penetration in dentin, along with the uptake and distribution of D-ribose within the extracellular and collagen matrices. 

Clinically, long-term resin–dentin bond strength is required. However, the degradation of collagen fibrils poses a significant problem, especially when the dentine is subjected to etching, leading to endogenous activation of MMPs. This in vitro study shows that dentin modifiers such as D-ribose crosslinker may be recommended in daily practice without involving an additional step or pre-treatments. Pre-treatments are generally uncontrolled suggestive of no control over the crosslinking effect. The results further reinforce the importance of MMP inhibition and collagen crosslinkers for the preservation of the integrity of the resin–dentin interface as a result of collagen crosslinking within the dentine fibrillary network. 

The limitations of this study should also be taken into consideration. First of all, the effect of dentin fluid and the presence of odontoblastic processes throughout most of the tubule length in clinical studies could not be found in vitro. Ideally, when an adhesive is tested in vitro, a clinical investigation should be conducted immediately to assess the clinical effectiveness of the adhesive. Next, further studies are needed to test more dental adhesives to demonstrate comparison between different materials. In addition, storage was done for 24 h; hence, there was more need for longer studies at differentiated time spans. Moreover, the adhesive was only tested in self-etch mode. Further investigations, including testing the same modified or unmodified adhesive in etch-and-rinse mode, are imperative for expanding the knowledge of ribose interaction. No comparisons of the ribose’s effect with other crosslinking agents were made. In addition, the smear layer was standardized using 600 SiC paper, which is clinically considered as a thin layer compared to a smear layer made by burs in vivo. Further studies could be performed with an accurate, safe and non-destructive method like micro-computed tomography (micro-CT) instead of SEM. Tests like cytotoxicity and nano-indentations including three-point bending should be researched in future clinical studies. 

## 5. Conclusions

Universal adhesive, using 1% or 2% RB, helped in maintaining dentin collagen scaffold and proved to be successful in improving wettability, proteases inhibition, and stability of demineralized dentin substrates.

## Figures and Tables

**Table 1:** Chemical composition of universal adhesive and instruction for material used.

Material and Manufacturer	Composition	pH	Instructions for Use
Prime&Bond active^TM^; Dentsply DeTrey GmbH, Konstanz, Germany	bisacrylamide 1 (25–50%), 10-methacryloxydecyl dihydrogen phosphate (10-MDP) (10–25%), bisacrylamide 2 (2.5–10%), 4-(dimethylamino)benzonitrile (0.1–1%), dipentaerythritol pentacrylate phosphate (PENTA) propan-2-ol (10–25%) water (20%)	2.5	apply adhesive, slight agitation (20 s), mild air-blowing (5 s), light-curing (20 s)

**Table 2:** Mean ± SD of micro-tensile bond strength (μTBS) in MPa in different groups after 24 h. In the rows, groups identified by different symbols are statistically different (*p* < 0.05). μTBS: micro-tensile bond strength. Mean ± SD for inhibition of MMP-2 and cathepsin-K activities 7 and 14 days. Comparison of MMP-2 and cathepsin-K activities (ng/mL) obtained with Human MMP-2 and cathepsin-K ELISA Kit system. Groups identified by different superscripts letter are statistically significantly different within each column. Mean ± SD of contact angle in different groups after treating with different concentrations of RB. Groups identified by different superscripts capital letters for different groups (shows differences between variables) are statistically different within each column.

Groups	μTBS	MMP-2		Cathepsin-K		Contact Angle
**Time**	**24 h**	**7 days**	**14 days**	**7 days**	**14 days**	**5 min**
**Control group**	36.21 ± 13.8 A	7.80 ± 1.7 A	11.10 ± 1.8 A	4.11 ± 2 A	5.7 ± 1.9 A	28.17 ± 8.1 C
**0.5% Ribose**	34.44 ± 5 B	4.10 ± 0.9 B	7.80 ± 1.4 B	2.90 ± 0.6 B	1.11 ± 2 A	8.650 ± 4.2 A
**1% Ribose**	39.44 ± 7.7 C	1.80 ± 1.2 C	4.40 ± 1.6 C	1.20 ± 0.3 C	0.51 ± 0.2 A	13.950 ± 3.9 A,B
**2% Ribose**	31.31 ± 6.7 D	0.90 ± 0.5 D	2.20 ± 0.7 D	0.40 ± 0.09 D	0.2 ± 0.1 A	22.025 ± 6.2 B,C

For each column, groups identified by different symbols are statistically different (*p* < 0.05). MMP-2: matrix metalloproteinases-2.

**Table 3:** Distribution of nanoleakage scores of the five groups of experimental adhesives at baseline. Extent of interfacial nanoleakage 0: 0%; 1: <25%; 2: 25–50%; 3: 50–75%; 4: >75%. Cochran–Mantel–Haenszel test (2 × 2 × 5).

Groups	Nanoleakage Score					
	0	1	2	3	4	*p*-Value
Control	0	30	5	25	40	*p* < 0.08
0.5% RB	5	25	10	45	25	*p* < 0.04
1% RB	30	10	15	30	15	*p* < 0.05
2% RB	0	10	10	35	45	*p* < 0.09

## Data Availability

The data presented in this study are available on reasonable request from the corresponding author.
